# Higher Risk of Non-Contact Anterior Cruciate Ligament Injury in the Non-Dominant Limb during Single-Leg Lateral Jump Landing in Active Individuals

**DOI:** 10.5114/jhk/205897

**Published:** 2025-11-19

**Authors:** Pinyada Warathanagasame, Miraj Subedi, Komsak Sinsurin, Jatuporn Suttiwong

**Affiliations:** 1 Physical Therapy Center, Faculty of Physical Therapy, Mahidol University, Salaya, Thailand.; 2 Hospital for Advanced Medicine and Surgery, Mandikhatar, Kathmandu, Nepal.; 3 Biomechanics and Sports Research Unit, Faculty of Physical Therapy, Mahidol University, Salaya, Thailand.; 4 Functional Outcome Measure Research Unit, Faculty of Physical Therapy, Mahidol University, Salaya, Thailand.

**Keywords:** biomechanics, injury risk, knee valgus, limb dominance

## Abstract

Non-contact anterior cruciate ligament injury is a common sports injury, for which biomechanical factors are considered important. Gender and limb dominance influence lower limb biomechanics. Therefore, this study aimed to investigate the effects of gender and limb dominance on trunk and lower extremity biomechanics in the frontal plane during single-leg lateral jump landing. A total of 20 active individuals (10 male and 10 female) were recruited to perform lateral single-leg jump landing. The study was conducted in a motion analysis laboratory. Ten Vicon™ cameras (200 Hz) synchronized with an AMTI force plate (1,000 Hz) were used to capture kinematic and kinetic data. A three-dimensional (3D) model of the trunk and lower extremities was constructed using Visual3D software. Average data of ground reaction forces (GRFs), frontal motion of the trunk and lower limb, and frontal joint moment of the lower limb were reported and analyzed. A two-way mixed ANOVA model (2 × 2, gender × side) was used to analyze the primary effect. The significance level of 0.05 was set for statistical analysis. Active females exhibited significantly higher peak vertical GRF (p = 0.01) than males. The non-dominant limb had a significantly higher knee valgus moment at the peak vertical GRF (p < 0.001), the knee valgus angle excursion (p = 0.03), and the hip abductor moment at the peak vertical GRF (p < 0.001). Both groups used similar frontal plane lower extremity control strategies. A significantly higher knee valgus excursion, hip abductor moment, and knee valgus moment at the peak vertical GRF phase were observed in the non-dominant limb. Therefore, the non-dominant limb appeared to have higher loading at the hip and knee joints during landing.

## Introduction

Anterior cruciate ligament (ACL) injury is the most common type of sports injury in athletes (Bram et al., 2021); 70% of ACL injuries involve non-contact mechanisms related to high-impact and pivot movements, such as side-step cutting and single-leg jump landing (Montalvo et al., 2019; Sanders et al., 2016). Previous studies have presented the risk factors of non-contact ACL injury during single-leg landing, including high ground reaction forces (GRFs) in the vertical and medial directions and a high knee valgus angle and moment (Imai et al., 2025; Montalvo et al., 2019; Nedergaard et al., 2020; Pfeifer et al., 2018).

The dominant effect is one factor that affects the differences in biomechanics between the lower extremities (Aizawa et al., 2018; Nakahira et al., 2022). Previous research has exhibited the difference in knee kinematics during a single-leg drop vertical jump and side-step cutting between the dominant and non-dominant sides. Female soccer players had higher knee valgus and internal rotation on the non-dominant side during landing (Nakahira et al., 2022), while in healthy participants, these were present during side-step cutting (Pollard et al., 2020). Nevertheless, another study about muscle fatigue during changes of direction reported higher knee valgus and tibial rotation on the non-dominant side (Hosseini et al., 2024); these findings are related to the high-risk biomechanics of non-contact ACL injuries (Larwa et al., 2021). However, some studies have suggested that the dominant limb showed a higher risk of knee injury during landing in volleyball athletes (Sinsurin et al., 2017, 2020). Currently, the effect of limb dominance on lower limb biomechanics remains controversial.

Females present a higher incidence rate of non-contact ACL injury than males, especially in high-impact rotational sports (Montalvo et al., 2019). This is attributed to differences in anatomical structure, the hormonal profile, and neuromuscular development (Mancino et al., 2024). Additionally, healthy females show higher peak vertical ground reaction force (vGRF) during single-leg lateral drop landing compared to males (Aizawa et al., 2018). This finding aligns with the study of Arundale et al. (2020) which reported greater knee valgus and poorer knee control during drop vertical jumps in females compared to males. Previous studies have focused on the influence of either gender or limb dominance as isolated factors (Hosseini et al., 2024; Nakahira et al., 2022; Sinsurin et al., 2017, 2020). When considering both gender and limb dominance, a higher incidence of non-contact ACL injuries has been observed in the non-dominant limb of females, whereas males have shown a higher incidence of non-contact ACL injuries in the dominant limb (Brophy et al., 2010). These findings have led to investigations into the biomechanical risk factors of non-contact ACL injuries, particularly how gender interacts with limb dominance. A better understanding of these biomechanics could help develop targeted prevention programs to reduce non-contact ACL injuries.

A previous study investigated the effect of gender and limb dominance on the GRFs during lateral single-leg jump landing (Aizawa et al., 2018). However, we believe that GRFs alone cannot explain the observed phenomena clearly. The examination of hip, knee, and ankle kinematics and kinetics, particularly in the frontal plane, could provide a better understanding of the biomechanical risks associated with non-contact ACL injuries. Furthermore, lateral trunk bending toward the stance limb influences the transition of the body’s center of body mass and affects lower extremity biomechanics during sports tasks (Hirohata et al., 2021; Houck et al., 2006). Therefore, the current study aimed to investigate the effects of gender and limb dominance on trunk and lower extremity biomechanics in the frontal plane during single-leg lateral jump landing. We hypothesized that female individuals would exhibit greater movement risks, such as increased knee valgus, compared to male individuals. In addition, significant differences in the trunk and lower limb biomechanics between the dominant and non-dominant limbs were expected to be observed during single-leg lateral jump landing.

## Methods

### Participants

A total of 20 healthy participants (10 male and 10 female) were recruited with a convenient sampling technique by advertising posters on social media. The active status included a moderate level of physical activity of at least 150–300 min per week or a vigorous level of physical activity of at least 75–150 min per week (Piercy et al., 2018). All active participants were male or female and aged 18–40 years. The exclusion criteria included participants who had trauma or surgery within three months before data collection, neuromuscular symptoms, balance deficit, serious injury of the lower extremity, knee instability of the ACL by two positives out of four tests (the anterior drawer test, the Lachman test, the pivot shift test, and the lever sign), and the body mass index (BMI) of more than 30 kg/m^2^ (Huang et al., 2016; Leblanc et al., 2015). The sample size estimation was calculated by G*power using the data of the peak medial GRF from the study of Aizawa et al. (2018). The mean (SD) values of the peak medial GRF in males and females were 48.3 N/kg (12.6) and 60 N/kg (6.6), respectively. Estimations with 80% power, 5% alpha level, and 20% missing data were calculated, and a sample of 10 subjects per group was required.

All participants were provided with the details of the study and signed an informed consent form before taking part. The research protocol was approved by the Mahidol University Central Institutional Review Board, Salaya, Thailand (approval code: MU-CIRB 2021/233.2212; approval date: 22 December 2021) before data collection.

### Measures

**Figure 1:**
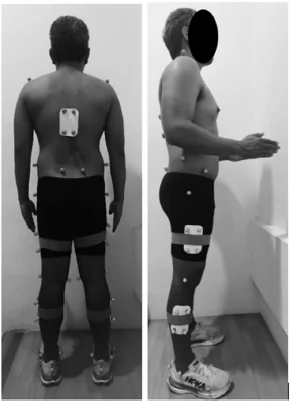
The position of reflective markers reference from the CAST model.

In this study, the dominant limb was identified by asking the participants which leg they preferred for kicking a ball (van Melick et al., 2017). Demographic data, leg length (measured from the anterior superior iliac spine [ASIS] to the medial malleolus), the Trendelenburg test results, and knee instability test performances were evaluated. The study was conducted in a motion analysis laboratory. A total of 10 cameras (Vicon™ Nexus, Oxford Metrics, UK), synchronized with a force plate (AMTI, USA), were used to capture kinematic and kinetic data. The sampling rates for the cameras and force plate were 200 and 1,000 Hz, respectively. A total of 26 reflective markers and five clusters of four reflective markers were attached to the participants based on the Calibrated Anatomical System Technique model (Cappozzo et al., 1996) (Figure 1). The positions of the 26 reflexive markers were on both sides of the acromion process, mid-point of the iliac crest, ASIS, posterior superior iliac spine, greater trochanter, medial and lateral femoral epicondyle, medial and lateral malleolus, posterior calcanei, distal head of the first and fifth metatarsals, and proximal head of the fifth metatarsals. Four clusters of four reflective markers were placed on the lateral aspect of the thighs, shanks, and spinal process at the T-10 level.

### Design and Procedures

**Figure 2:**
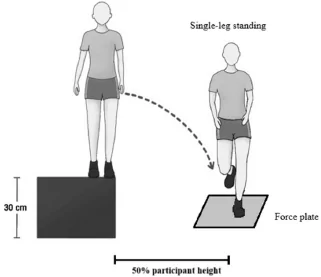
Laboratory setting for lateral single-leg drop jump landing for the left leg.

This study utilized a cross-sectional design to investigate trunk and lower extremity movements. Before undergoing the lateral single-leg jump-landing tests, participants completed a 5-min warm-up consisting of jogging, followed by practice of jump-landings to familiarize themselves with the testing procedure. Lateral single-leg jump landing was performed from a box of 30-cm height, and the jump distance was 50% of the participant’s height (Figure 2) (Aizawa et al., 2018; Hirohata et al., 2021). Participants were instructed to land on the center of the force plate, and the dominant and non-dominant limbs were tested in random order. The successful trial of jump landing was a condition of the ability to land with the tested limb without loss of balance or contact with the floor of the non-tested limb. Participants were required to maintain a single-leg stance for at least 3 s during landing. Three successful repetitions were recorded for each side, with a 5-min rest interval allocated between limb tests.

### Data Acquisition

**Figure 3:**
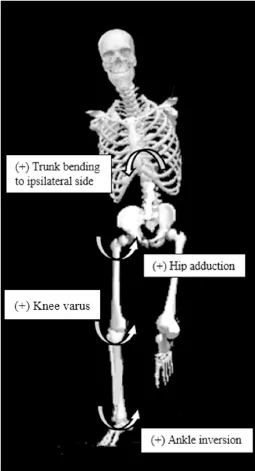
Angular motion of the trunk and lower limb joints in the frontal plane; positive value represents the trunk bending to the ipsilateral side, hip adduction, knee varus, and ankle inversion.

After data collection, the markers’ positions and GRFs were filtered with a 4^th^ order zero-lag low-pass Butterworth filter at a cut-off frequency of 6 and 50 Hz, respectively. A three-dimensional (3D) model of the trunk and lower extremities was constructed using Visual3D software (C-motion Inc., Germantown, USA). The movement directions of the positive and negative values are shown in Figure 3. Positive values indicate the trunk bending to the ipsilateral side, hip adduction, knee varus, and ankle inversion, while negative values indicate the trunk bending to the contralateral side, hip abduction, knee valgus, and ankle eversion.

The initial contact (IC) phase was defined as the moment when the foot first contacted the force plate, with the vGRF exceeding 10 N. The landing phase started at the IC and was 300 ms after the foot contact. Subsequently, the parameters of the current study were extracted, including the peak medial GRF, frontal joint angles at the peak vGRF, and movement excursions. The movement excursion was the angular displacement from the IC to the peak vGRF. All parameters were averaged across three successful trials and registered for further analysis.

### Statistical Analysis

This study used the SPSS statistics version 23 (BMI, USA) for statistical analysis. The Shapiro-Wilk test was performed to check the data distribution, and all variables presented a normal distribution. A two-way mixed ANOVA model (2 × 2, gender × side) was applied to analyze the primary effect. The significance level was set at 0.05 for all statistical tests.

## Results

The characteristics of the 20 active participants are shown in Table 1. The mean age and BMI showed no significant difference between the male and female groups.

**Table 1:** Participants’ characteristics. A moderate level of physical activity included walking or body weight training at least 150–300 min per week. A vigorous level of physical activity included jogging, running, playing soccer, swimming or weight training at least 75–150 min per week.

Variables	Males (n = 10)	Females (n = 10)
Age (year) (mean ± SD)	25.67 ± 5.03	26.50 ± 6.26
BMI (kg/m^2^) (mean ± SD)	21.97 ± 1.46	20.01 ± 2.21
Dominant side (number, %) - Left - Right	3 (30%) 7 (70%)	0 10 (100%)
Physical activity level (number, %) - Moderate level - Vigorous level	2 (20%) 8 (80%)	4 (40%) 6 (60%)

Tables 2 and 3 show no significant differences in the interaction effect of gender and the side. A significant difference in peak vGRF was observed (*p* = 0.01) (Table 2). The main side effects were significant differences in the knee valgus moment at the peak vGRF (*p* < 0.001), the knee valgus angle excursion (*p* = 0.03), and the hip abductor moment at the peak vGRF (*p* < 0.001).

**Table 2:** Angular excursions of trunk and lower limb joints in the frontal plane during lateral single-leg jump landing (mean ± SD).

Variables	Males		Females		Gender effect F, *p*-value	Side effect F, *p*-value	Interaction effect F, *p*-value
	Dominant side	Non-dominant side	Dominant side	Non-dominant side			
Trunk lateral bending excursion (°)	5.19 ± 3.24	6.14 ± 3.81	2.26 ± 3.63	4.78 ± 2.38	4.19, 0.05	4.11, 0.05	0.85, 0.37
Hip adduction excursion (°)	10.96 ± 5.60	12.09 ± 6.33	8.90 ± 13.02	12.15 ± 4.24	0.17, 0.68	0.97, 0.33	0.23, 0.64
Knee valgus excursion (°)	3.15 ± 1.70	3.95 ± 1.70	2.67 ± 2.73	3.38 ± 2.61	0.38, 0.55	5.33, 0.03*	0.02, 0.89
Ankle eversion excursion (°)	1.97 ± 5.90	2.17 ± 3.92	1.38 ± 4.84	2.93 ± 5.36	0.002, 0.96	0.62, 0.44	0.38, 0.55

* indicates significant difference (p-value < 0.05)

**Table 3:** GRFs and joint moments at peak vGRF during lateral single-leg jump landing (mean ± SD).

Variables	Males		Females		Gender effect F, *p*-value	Side effect F, *p*-value	Interaction effect F, *p*-value
	Dominant side	Non-dominant side	Dominant side	Non-dominant side			
Peak vGRF (N/kg)	3.55 ± 0.52	3.45 ± 0.42	4.18 ± 0.62	3.99 ± 0.55	8.47, 0.01*	3.26, 0.08	0.25, 0.62
Peak medial GRF (N/kg)	0.30 ± 0.10	0.26 ± 0.06	0.32 ± 0.08	0.29 ± 0.08	0.99, 0.33	2.97, 0.10	0.04, 0.84
Hip abductor moment at peak vGRF (Nm/kg)	-0.57 ± 0.29	-0.75 ± 0.19	-0.58 ± 0.26	-0.90 ± 0.09	1.24, 0.28	22.11, <0.001*	1.61, 0.22
Knee valgus moment at peak vGRF (Nm/kg)	-0.14 ± 0.21	-0.31 ± 0.12	-0.13 ± 0.11	-0.30 ± 0.09	0.11, 0.74	17.30, <0.001*	0.002, 0.96
Ankle evertor moment at peak vGRF (Nm/kg)	-0.09 ± 0.44	0.01 ± 0.04	0.01 ± 0.05	0.004 ± 0.04	0.51, 0.48	0.48, 0.49	0.74, 0.40

* indicates significant difference (p-value < 0.05)

## Discussion

This study aimed to investigate the effects of gender and limb dominance on different biomechanics in the frontal plane of the trunk and lower extremities during lateral single-leg landing. The findings showed that the interaction effect of gender and limb dominance did not significantly affect variables considered in the current study. Aizawa et al. (2018) observed a significant interaction between gender and limb dominance in the vGRF at the peak medial GRF during lateral single-leg landing. They suggested that, in male individuals, the dominant limb exhibited a better capacity to absorb the frontal impact force than the non-dominant limb. The vGRF at the peak medial GRF was similar in both limbs in female individuals (Aizawa et al., 2018). Differences in time events and the research setting of the jump-landing tests could explain the discrepancy between the findings of the Aizawa et al.’s (2018) study and the current research.

The main effect of gender had a significant effect on the peak vGRF, and a significant main effect of limb dominance was observed on hip and knee biomechanics. Owing to the inconsistencies in non-contact ACL injury rates reported in previous studies, this finding may provide insight into the differences in frontal plane movement biomechanics during lateral single-leg landings, which could be associated with the risk of non-contact ACL injury in each limb. A higher rate of non-contact ACL injury has been reported in female athletes (Chia et al., 2022; Montalvo et al., 2019). In the current study, female individuals exhibited higher peak vGRF during landing than males. This finding aligns with previous studies (Aizawa et al., 2018; Arundale et al., 2020) which showed a high peak vGRF during single-leg vertical jump landing and side jump landing. However, no significant differences in frontal joint mechanics were observed between the male and female groups in the present study. High peak vGRF is associated with increased knee joint loading and ACL stress during dynamic movement, which increases the risk of ACL injuries (Donelon et al., 2020). Previous studies have suggested that high peak vGRF is linked to reduced hip and knee angular excursions (Arundale et al., 2020; Hirohata et al., 2021); however, the current study observed no significant gender differences in the frontal plane hip and knee angles. Despite the higher peak vGRF observed in females, it appears that males and females used a similar control strategy in the frontal lower extremity movement. Future research may explore lower extremity control in the sagittal and horizontal planes to provide a more comprehensive understanding of the biomechanical differences between genders.

The non-dominant limb was reported to present higher biomechanical risks for ACL injury than the dominant limb, including decreased knee flexion, increased hip flexion, adduction, and internal rotation at the IC (Hosseini et al., 2024; Nakahira et al., 2022). In the current study, significantly higher hip abductor and knee valgus moments at the peak vGRF phase were observed in the non-dominant limb. This suggests that the non-dominant limb had greater loading at the hip and knee joints during landing. Furthermore, the non-dominant limb landed with a higher knee valgus excursion than the dominant limb. This finding could indicate that, in active individuals, the non-dominant limb may be at a higher risk of non-contact ACL injury owing to poor control and high loading of the knee valgus excursion and the knee valgus moment. This study supports previous research which demonstrated that the non-dominant limb contacted the ground with a higher knee valgus and internal rotation angles during single-leg drop vertical jumps and side-step cutting compared with the dominant limb (Nakahira et al., 2022; Pollard et al., 2020). Moreover, a higher moment of the hip abductor observed in the non-dominant limb may reflect compensatory movement. To mitigate the risk of injury associated with an excessive knee valgus excursion and joint moments, greater activation of the hip adductor muscles is required to stabilize the femur or control hip adduction, as suggested by previous research (Donelon et al., 2020; Hosseini et al., 2024). Although the biomechanical variables observed in this study showed relatively small differences in magnitude between limbs, these differences might have significant implications in real-world sports and practice scenarios where physical and mental demands are substantially higher.

In the non-dominant limb, the higher-risk biomechanics in the frontal plane of the hip and knee joints suggest an increased risk of ACL injuries during dynamic movement in active individuals. In contrast to previous research (Nakahira et al., 2022), Sinsurin et al. (2017, 2020) suggested that the dominant limb showed a higher risk of knee injury during landing. However, Sinsurin et al.’s (2017, 2020) studies were conducted on volleyball athletes, while Nakahira et al. (2022) observed a greater knee valgus at the IC in the non-dominant limb in soccer players. Different sport specific movement patterns and training likely influence lower limb biomechanics. The current study investigated active individuals who did not participate in any specific sport training. Therefore, the current findings should be carefully applied to other populations. Further studies are suggested to investigate specific athletes involved in various sports, particularly high-impact rotational sports, where movement patterns significantly differ. Injury prevention programs should emphasize movement control in the frontal plane, particularly hip adduction and the knee valgus excursion in the non-dominant limb in active individuals. Strength training and correct landing techniques should be implemented to prevent excessive hip adduction and knee valgus during single-leg lateral landing. Male and female participants exhibited similar strategies for movement control in the frontal plane, suggesting that health professionals can apply the same injury prevention program for both genders.

## Conclusions

The interaction effect of gender and limb dominance did not significantly affect the variables considered in the current study. The main effect of gender was significant on the peak vGRF, and a significant main effect of limb dominance was observed on hip and knee biomechanics. In the current study, female individuals exhibited a higher peak vGRF during landing than males. However, the current study found no significant gender differences in the biomechanics of the frontal plane of the trunk, the hip, the knee, and the ankle. Despite the higher peak vGRF observed in females, it appears that males and females employed similar frontal plane lower extremity control strategies. A significantly higher knee valgus excursion, hip abductor moment, and knee valgus moment at the peak vGRF phase were observed in the non-dominant limb. Therefore, the non-dominant limb appeared to have higher loading at the hip and knee joints during landing in the current study.
